# Health Related Quality of Life (HRQOL) and its correlation with academic performance of medical students

**DOI:** 10.12669/pjms.35.1.147

**Published:** 2019

**Authors:** Shahid Sarwar, Abdul Aleem, Muhammad Arif Nadeem

**Affiliations:** 1 Shahid Sarwar, MBBS, FCPS (Med) FCPS (Gastroenterology) MCPS-HPE. Associate Professor, Services Institute of Medical Sciences Lahore, Pakistan; 2 Abdul Aleem, MBBS. Post-Graduate Resident Gastroenterology & Hepatology, Services Institute of Medical Sciences Lahore, Pakistan; 3 Muhammad Arif Nadeem, MBBS, FCPS (Medicine). Professor of Medicine, Services Institute of Medical Sciences Lahore, Pakistan

**Keywords:** Academic performance, Health related quality of life, Mental health, Physical health

## Abstract

**Objective:**

To determine health related quality of life (HRQOL) of medical students and its correlation with their academic performance.

**Methods:**

Cross sectional study at Services Institute of Medical Sciences, included students of 4^th^ and final year MBBS, who filled SF-36 proforma of HRQOL. Scores of 8-domains and of physical component and mental component summary were determined. Marks in all professional examinations were used to stratify students as high performers (≥ 70% marks) and average performing students (< 70%). HRQOL scores was correlated with academic performance using unpaired student’s *t*-test.

**Results:**

Among 267 students included, mental health score (56.2±21.3) was lower than physical health component score (69.03±18.5). Role limitation due to emotional health (RE) (44.81), Vitality (VT) (54.19) and general health perception (GH) (58.89) had lower scores among 8-domains of questionnaire. Female students had significantly lower scores in role limitation due to emotional problems (p value <0.04), vitality (<0.05), bodily pain (p value <0.05) and general health perception (p value<0.03) than male students. Physical health and role limitation due to physical health domains were better in high performing students.

**Conclusion:**

Mental health of medical students is suboptimal, especially among female students. Students with better physical health have better academic performance.

## INTRODUCTION

The term quality of life was first introduced in social sciences after Second World War. It was the consequence of realization that good life is more valuable than financial well-being which was always considered a bench mark for standard of living.^1^ The WHO defines Quality of Life as “an individual’s perception of their position in life in the context of the culture and value systems in which they live and in relation to their goals, expectations, standards and concerns”.^2^ It is a broad ranging concept affected in a complex way by the person’s physical health, psychological state, personal beliefs, social relationships and their relationship to salient features of their environment.^3^ In other words quality of life is the outcome of individual’s perceptions. Health related quality of life (HRQOL) represents the influence of health status, medical treatment and health policies on these perceptions of well-being.^4^

Medical students are a highly educated population group, who encounter multiple emotions during transformation from insecure student to young knowledgeable physician.^5^ During their five years stay in medical college and especially after introduction to clinical settings in senior years, they encounter loss of control, illnesses and helplessness while dealing with patients and their diseases, sometimes resulting in worsening depression and anxiety. It can lead to deteriorating mental health of students. Multiple factors can exaggerate this situation such as academic overload,^6^ contact with diseases^7^ and death and ever expanding medical curriculum.^8^ Anxiety, depression and worsening stress is on rise among medical students.^9^

Studies have identified a strong link between these factors responsible for poor health related quality of life and academic performance of students.^10^ Academic performance of a student reflects his learning. Being groomed to practice a sensitive profession like medicine, his personal and professional development for future practice is depicted in performance of examinations. Studies have shown that academic performance of a student can predict his professional competence later.^11^

Multiple factors can affect his academic performance like, learning environment, instructional strategies being adopted at institution, curricular overload and attitude of faculty. But does health related quality of life effect students learning is still far from being fully explored.

Objective of our study was to determine the health related quality of life of students of medical college and to correlate it with their academic performance.

## METHODS

This cross-sectional study was conducted at Services Institute of Medical Sciences Lahore in August 2018. Approval was obtained from the institutional review board. Purposive non-probability sampling was done. Students of 4^th^ year and final year, having at least four years of professional learning and been through multiple professional examinations were included in study. Informed consent was taken from the participants. Students not willing to participate and those with pending result of professional examination were excluded. Students filled the study proforma in a dedicated session of one hour.

Instrument used for evaluation of HRQOL was Short Form-36 (SF-36), a 36 item questionnaire developed in Medical Outcome Study (MOS) for measuring health related quality of life.^12^ It measures quality of life across eight domains, which are both physically and emotionally based. These domains are Physical Functioning (PF), role limitation due to physical health (RP), role limitation due to Emotional Problems (RE), Vitality (VT), General Mental Health (MH), Social Functioning (SF), bodily pain (BP) and General Health perception (GH). The SF36 has been widely validated for numerous professions and patient groups.^13^

Once filled, an aggregate percentage score is produced for each of eight domains measured by SF-36. It is done in two step process. Each of the question responses is related to a different pre-coded numeric value. The response to each question is translated into raw score from 0 to 100, with 0 representing a very low level quality of life (QoL) and 100 depicting a very positive response to the item. In second step, we took these translated item scores and determined the score for each of the eight domain by adding scores of items related to a domain and dividing it by number of items used. For domain of PF, we added scores of item no 3, 4, 5, 6, 7, 8, 9, 10, 11 and 12 and divided it by 10. Similarly we added scores of item 13, 14, 15 and 16 and divide it by 4 to determine score of RP, scores of item 17,18,19 gave score of RE, item 23, 27, 29 and 31 gave score for VT, item 24, 25, 26, 28 and 30 calculated score for MH, addition of item 20 and 32 and dividing by 2 produced score for SF, 21 and 22 items for BP and item 1, 33, 34, 35 and 36 gave score for GH. We also calculated Physical component Summary(PCS) depicting physical QoL by averaging scores of PF, RP, BP and GH and Mental Component Summary(MCS) representing mental QoL by determining average of SF, RE, MH and VT. We used criterion based interpretation of SF-36 to correlate it with academic performance of students.

Academic performance was determined by adding percentage marks of all professional examinations taken by students and dividing it by number of examinations. Students with percentage of 70% or above were classified as high performers while those with less than 70% percentage in aggregate of all professional examinations were labeled as average performers.

### Statistical Analysis

Data was analyzed using SPSS® 20. Variable were expressed as mean ± standard deviation (SD) or in percentage where appropriate for normally distributed variables while median and interquartile range (IQR) for variables not normally distributed. Shapiro-wilk test was used for checking whether variables were normally distributed or not. We determined Cronbach’s alpha to determine internal consistency of SF-36 and value above 0.7 was considered satisfactory for study group.

Eight domains measured by SF-36, Physical Component Summary (PCS) and Mental Component Summary (MCS) were analyzed for association with academic performance using unpaired student’s t test for normally distributed variables while Mann-Whitney’s test for non-parametric variables.

## RESULTS

Total of 267 students participated in study, 184(68.9%) girls and 83 (31.1%) boys. Student participation in survey was 91.3% (137/150) in final year and 86.6% (130/150) in 4^th^ year class. Data collected from SF-36 was checked for internal consistency by determining Cronbach’s alpha, which was 0.918, suggesting excellently consistent data. Apart from PF, data of other domains of quality of life was normally distributed. Scores of students were better in domains of PF (mean 82.94 ±20.09) and BP (mean 73.73±34.6) while mean scores in RE (44.81), VT (54.19) and GH (58.89) were relatively on lower side. RF (60.58), MH (62.20) and SF (63.71) domains had average values. Mean score for physical component summary was 69.03 (±18.49) and for mental component summary it was 56.23 (±20.5) suggesting poor state of emotional health in our students.

Comparison of male and female students in scores across different domains of quality of life is shown in Table-I. Scores of Role limitation due to emotional problems (RE) (p value 0.04), Vitality (VT) (p value 0.05), bodily pains (BP) (p value 0.05) and general health perceptions (GH) (p value 0.03) were significantly better in male students. Male students had better physical component summary (PCS) scores, 73.03(±15.15) as compared to 67.23(±18.8) in female students (p value 0.01). Mental component summary scores were not significantly different, 59.64(±21.15) in male as compared to 54.69(21.35) in female students (p value 0.08).

**Table-I:** Effect of Gender on domains of quality of life

Domains of QOL	Male students score (Mean ± SD)	Female students score (Mean ± SD)	P value
Physical functioning (PF)	84.93 (19.0)	82.03 (20.1)	0.27
Role limitation due to physical health (RP)	66.86 (37.06)	57.74 (39.52)	0.07
Role limitation due to emotional health (RE)	52.61 (43.5)	41.3 (42.35)	0.04
Energy/Fatigue (VT)	57.46 (18.2)	52.7 (18.9)	0.05
Emotional well-being (MH)	65.1 (20.1)	60.8 (20.1)	0.11
Social functioning (SF)	63.4 (24.5)	63.8 (24.05)	0.88
Pain (BP)	77.74 (22.7)	71.92 (22.5)	0.05
General health perception (GP)	62.59 (17.7)	57.2 (20.17)	0.03
Physical component summary	73.03 (15.1)	67.23 (18.8)	0.01
Mental component summary	59.64 (21.1)	54.69 (21.3)	0.08

Students of 4^th^ year and final year were comparable in all domains of quality of life except general health (GH) which was better for final year students, 62.29(±18.97) vs 4^th^ year score of 55.30 (±19.61) (p< 0.003).

On reviewing academic performance only 38(14.2%) students had cumulative academic scores of 65% or less, scores were between 65% to 70% in 66 (24.7%) students, 90 (33.7%) students had scores between 70% to 75% and 73 (27.3%) students had examination scores above 75%. As per our bench mark for performance status, 162 (60.7%) students were high performers with examination scores ≥ 70% while 105 (39.3%) were average performing students.

In order to identify effect of different domains of quality of life on academic performance, we compared high performing and average performing students as shown in Table-II. High performing students had better scores in most of SF-36 domains but difference was statistically significant only in domains of Physical Functioning (PF) and role limitation due to physical health (RP), both better in high performing students, 85.03(±17.12) mean score in PF as compared to 79.71 (±23.6) in average performing students (p value 0.03) and 64.5(±37.4) for high performers in RP as compared to 54.52(±40.5) of average performing students (p value 0.04).

**Table-II:** Domains of quality of life and academic performance.

Domains of QOL	High performers mean± SD	Average performers mean ± SD	P value
Physical functioning PF	85.03 (17.12)	79.71 (23.6)	0.03
Role limitation due to physical health RP	64.5 (37.4)	54.52 (40.54)	0.04
Role limitation due to emotional problems RE	46.5 (43.06)	42.2 (42.9)	0.42
Energy/fatigue VT	54.96 (18.0)	53 (20.06)	0.40
Emotional well-being MH	62.41 (19.6)	61.86 (21)	0.82
Social functioning SF	65.20 (23.7)	61.42 (24.7)	0.21
Pain BP	74.95 (21.6)	71.85 (24.3)	0.27
General health perception GP	58.14 (18.9)	60.04 (20.4)	0.43
Physical component summary PCS	70.65 (17.7)	66.53 (19.4)	0.07
Mental component summary MCS	57.27 (21.2)	54.62 (21.6)	0.32

## DISCUSSION

During last few decades, education has moved from behaviorism to constructivism due to understanding that instead of using examinations and grades to promote learning, focus should be on helping student in constructing his knowledge through self-directed learning.^14^ This transformation is further augmented by evolution of social learning theory which says that learning does not occur in isolation but is effected by learner’s environment and his sense of well-being.^15^

Impact of quality of life on learning of student is now being given due importance. Paro HB, et al identified that quality of life of students worsens over time during their stay in institution as it was lower in students of 4^th^, 5^th^ and 6^th^ year as compared to those in 1^st^ year of learning.^16^

We have observed lower scores in aspects of quality of life related to mental health as compared to physical component scores in our study. In a study of 165 medical students and 93 doctors, prevalence of depression was 28.6% and that of anxiety 28.7%.^5^ This impaired mental status may continue from student life to post-graduate training and may result in early burn-out in physician’s practical life. Cecil J, et al in a study of behaviors leading to burn out in medical students noted that 55% students report high level of emotional exhaustion, 34% experienced de-personalization and 46.6% had low level of personal accomplishment. Factors responsible for mental impairment were lack of physical activity, year of study and female gender.^17^ Encouragement of healthier life style and promotion of extra-curricular activities can help in improving mental health of students.

This effect is more pronounced in female students. Backovic DV, et al concluded that female students suffer more stress and burnout especially during attending autopsy and interaction with patients in clinical training.^18^ Female participants of our study had poor scores in role limitation due to emotional problems, vitality, bodily pains, general health perception and physical component summary scores as compared to male students. It brings forth need to identify root cause of poor status of these domains in female students and taking measures for improvement to aid their learning.

We have identified significant association between physical health related quality of life and academic performance of students, though high performing students had higher scores in most mental health domains of HRQOL as well. In a study of 78 medical students of Sri Lanka, quality of life scores were lower before examination as compared to scores after examination. It is understandable that examination adds to mental and physical stress. In this study students with better quality of life had superior performance in examination as well.^19^ McIsaac JL, et al in a study of elementary school students concluded that students with unhealthy lifestyle behaviors were more likely to have poor academic performance.^20^ Studies have identified genetic markers to explain complex association of poor health with lower academic achievements.^21^

We have only included 4^th^ and final year medical students. Inclusion of students of 1^st^ to 3^rd^ year may have made our results more generalizable. Another limitation can be timing of data collection as we conducted this survey close to professional examination and quality of life score can vary in relation to proximity to examination. Despite these limitations, our study has enabled us to highlight issues related to quality of life of our students, an aspect still largely unexplored in our region. It also guides us to conduct further research for in-depth study aimed at identifying measures to improve quality of life of medical students as this will facilitate better academic performance and brilliance in professional career.

## CONCLUSION

Mental health of medical students is suboptimal as compared to physical health especially among female students. Students with better physical health have better academic performance.

### Author’s contribution

**SS** conceived, designed, did statistical analysis and manuscript writing.

**SS and AA** did data collection and manuscript review.

**MAN** did manuscript review and final approval.
